# Tuberculous pseudoaneurysm of the renal artery: A rare manifestation of extra-pulmonary tuberculosis

**DOI:** 10.1016/j.eucr.2025.102939

**Published:** 2025-01-13

**Authors:** Sikai Song, Martin Hofmann, Herbert Ruckle, Forrest Jellison, Muhannad Alsyouf

**Affiliations:** Department of Urology, Loma Linda University, Loma Linda, CA, USA

**Keywords:** Mycotic pseudoaneurysm, Extrapulmonary tuberculosis, Nephrectomy

## Abstract

Mycotic pseudoaneurysms are rare dilations of the arterial wall caused by infection. We present a case of a 62-year old man with disseminated tuberculosis and a large mycotic pseudoaneurysm involving the main renal artery and vein. Despite being on appropriate rifampin, isoniazid, pyrazinamide, and ethambutol (RIPE) therapy, the pseudoaneurysm grew with increasing concern for potential rupture. The patient subsequently underwent an open right simple nephrectomy with resection of the pseudoaneurysm, partial resection of the inferior vena cava, and reconstruction.

## 1 Introduction

Pseudoaneurysms are rare and occur as a sequela of vessel wall destruction leading to weakening of the vessel and subsequent dilation. Unlike true aneurysms which are contained by all three layers of the arterial wall, a pseudoaneurysm is a locally contained hematoma that does not contain any layers of the arterial wall, bounded instead by products of the clotting cascade.^1^ While pseudoaneurysms are most commonly due to iatrogenic causes following endovascular procedures and vascular anastomotic complications, they can also be caused by trauma, infection, or inflammatory processes.^1^

Infectious pseudoaneurysms are a rare entity. Also known as “mycotic pseudoaneurysms”, they occur predominantly by hematogenous spread from bacterial or fungal infections. The most commonly involved arteries of mycotic pseudoaneurysms are the aorta, femoral, splanchnic, and cerebral arteries.^2^ Mycotic aneurysms of the renal artery are rare, with only 20 cases reported in the literature that occurred following renal transplantation due to Candida species.^3^ Mycotic aneurysms of the renal artery due to tuberculosis infection, or tuberculous pseudoaneurysm of the renal artery, are even rarer, with only one known prior case report published in 1976.^4^

We describe a case of a 62-year old man with disseminated tuberculosis complicated by Pott's Disease of T12-L1 with spinal epidural abscess and a large tuberculous pseudoaneurysm of the right renal artery involving the right renal hilum. The patient subsequently underwent an open right nephrectomy with en-bloc resection of the pseudoaneurysm, partial resection of the inferior vena cava (IVC), and reconstruction with the surgical technique detailed in this report.

## 2 Case presentation

A 62-year old male with limited access to routine health care presented to an ambulatory urgent care clinic for severe back pain and bilateral lower extremity weakness. Evaluation was notable for a creatinine of 3.7 mg/dl (unknown baseline) and a Computed Tomography (CT) chest demonstrated spiculated lung nodules with diffuse bilateral pulmonary micronodules in a miliary distribution suggestive of tuberculosis. Abdominal imaging demonstrated a 4 cm thoracic spinal abscess with significant osseous destruction of the lower thoracic spine. In addition, imaging revealed moderate left hydroureteronephrosis with a thickened bladder wall and a non-specific 7.4 cm right hilar mass with an atrophic right kidney. An MRI abdomen was obtained which demonstrated a large vascular lesion in the right renal hilum consistent with a pseudoaneurysm. A Lasix washout renogram subsequently showed a differential function of 6 % right kidney. Urine and sputum cultures demonstrated acid-fast bacilli (AFB), confirming the diagnosis of disseminated tuberculosis.

Patient subsequently underwent an urgent T11/12 laminectomy and a left percutaneous nephrostomy tube placement for renal decompression and preservation. Endovascular management of the pseudoaneurysm was not recommended due to infectious etiology, as well as the large size of the pseudoaneurysm near the great vessels. After an extensive discussion about management options and shared decision-making, the deconditioned patient proceeded with medical management of disseminated tuberculosis prior to consideration for surgical intervention for mycotic pseudoaneurysm. Rifampin, isoniazid, pyrazinamide, and ethambutol (RIPE) therapy was initiated, and the patient was discharged home with plan for repeat imaging to evaluate pseudoaneurysm after completion of treatment.

**Fig. 1:**
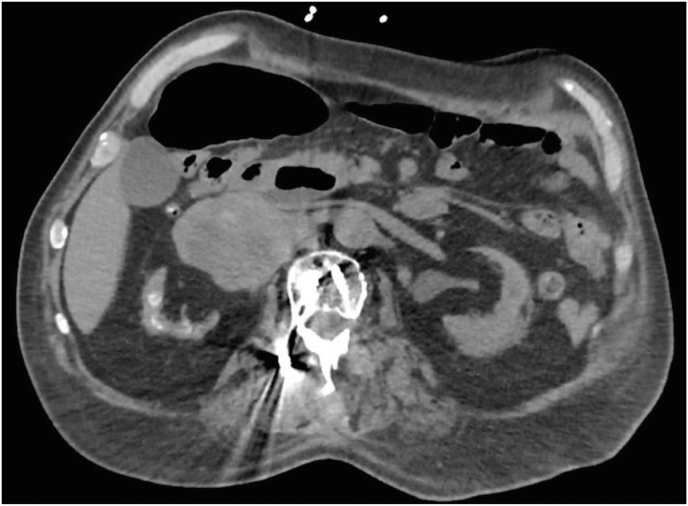
CT Abd/Pelvis demonstrating an 8.4 cm right renal artery pseudoaneurysm and renal atrophy.

However, the patient re-presented back to the emergency room two weeks later with generalized weakness. Repeat cross-sectional imaging demonstrated an enlarging pseudoaneurysm of the right renal artery now measuring 8.4 cm (Fig. 1). Given the concern for potential rupture of expanding pseudoaneurysm in a non-functioning kidney despite RIPE therapy, the decision was made to proceed with open nephrectomy and resection of mycotic pseudoaneurysm.

### 2.1 Surgical management

The patient underwent an open right simple nephrectomy with resection of the right renal artery pseudoaneurysm, and partial resection of the IVC. The patient was positioned supine and a midline incision was created just below the xiphoid process to 2 cm below the umbilicus. The Omni retractor was used to retract the body walls. An incision was made near the white line of Toldt and the right colon was reflected medially. The duodenum was noted to be adherent to the pseudoaneurysm capsule and required meticulous sharp dissection to kocherize the duodenum. The inferior vena cava was exposed and the pseudoaneurysm was identified. This was noted to also be adherent to the inferior vena cava at the level of the renal vein ostium. A split-and-roll technique was used expose the vena cava cephalad and caudad to the aneurysm and the lumbar veins were clipped and divided. The right gonadal vein was ligated with a 2-0 silk tie and transected at its origin. The interaortocaval region was dissected to identify the right renal artery at its origin from the aorta and suture-ligated with two 0-silk ties proximal to the pseudoaneurysm.

**Fig. 2:**
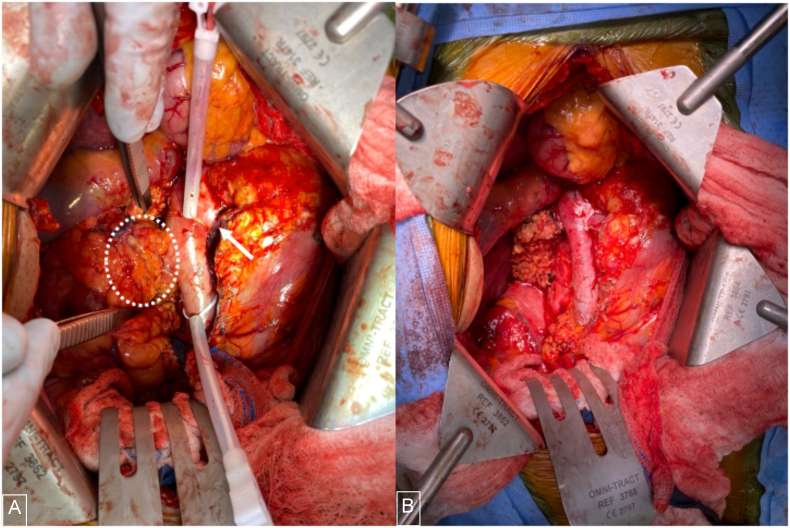
**(A)** Right pseudoaneurysm (dotted circle) and kidney with suprarenal and infrarenal inferior vena cava vascular control (white arrow demonstrates right renal artery identified in the interaortocaval space) (B) Surgical bed after right nephrectomy, en-bloc resection of pseudoaneurysm, and resection/reconstruction of the vena cava.

Attention was then turned to the hilar pseudoaneurysm which extended medially and involved the posterior portion of the IVC. Proximal and distal control of the IVC was achieved using Rummel tourniquets and umbilical tape. The pseudoaneurysm was then meticulously dissected and freed from the posterior vena cava. The Ligasure device was used to mobilize the kidney superiorly, laterally, and posteriorly. The right adrenal gland was spared. The right ureter was clipped and divided. Once the kidney and pseudoaneurysm were completely mobilized, the Rummel clamps were tightened and a 15# blade was used to transect the right renal vein at the end of its ostia. The specimen was then removed from the field and sent for permanent pathology (Fig. 2).

The IVC was then reconstructed using 4–0 prolene in a running fashion. Heparinized saline was injected into the lumen of the cava and the inferior tourniquet was released to remove any clots. After ensuring hemostasis, bowel was returned to anatomic position and the incision subsequently closed.

**Fig. 3:**
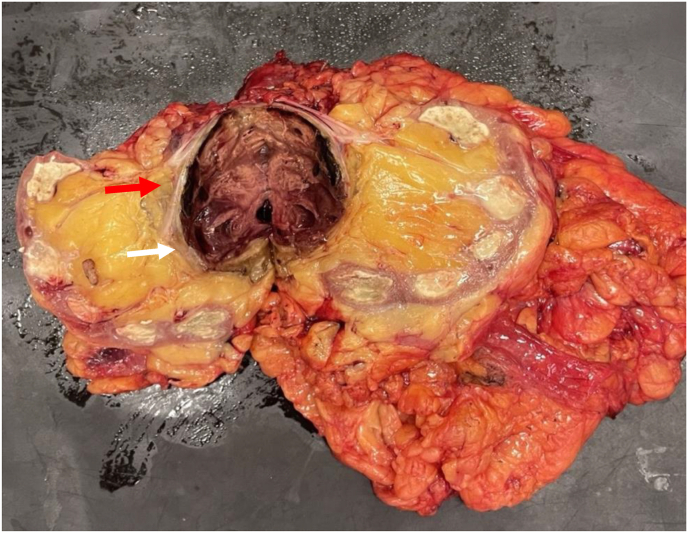
Gross specimen after bisection demonstrating the large mycotic aneurysm of the renal artery (white arrow), vein, and edge of inferior vena cava (red arrow).

Post-operatively, the patient recovered well and was discharged on postoperative day 14 with serum Cr level of 2.3. Pathology evaluation of the right kidney and pseudoaneurysm demonstrated end-stage kidney with old necrosis bordered by fibrosis with no granulomatous inflammation identified (Fig. 3).

## 3 Discussion

*Mycobacterium tuberculosis* primarily infects the lungs, but infection by hematogenous, lymphatic, or direct spread in other tissues and organs systems can also occur. An estimated 15 % of all infections worldwide occur at extrapulmonary sites, with or without pulmonary symptoms.^5^ Vascular manifestations of extra-pulmonary tuberculosis are rare, with few case reports of arterial tuberculosis published in the literature, including only three known cases of stenosing tuberculous arteritis of the renal artery.^6^ While there have been several case reports on mycotic pseudoaneurysms of the aorta, there is only one known case report of arterial tuberculosis manifesting as a pseudoaneurysm of the renal artery in the English literature.^4^

The majority of reported cases of tuberculous pseudoaneurysms occur in the thoracic and abdominal aorta with concomitant vertebral tuberculosis.^7^ Tuberculous pseudoaneurysms form through several proposed mechanisms, most commonly due to direct invasion and spread from adjacent lymph nodes, abscess, and bony tuberculosis.^7^ The tuberculosis-affected tissue nearby can cause direct injury to the full thickness of the vessel wall, which causes hemorrhage and perivascular hematoma in connection to the lumen of the aorta. This perivascular hematoma is contained by fibrosis that forms around the hematoma, hence the term “pseudoaneurysm”. In our patient, it is most likely that tuberculosis spread directly from the adjacent spinal abscess to the renal artery, leading to the presentation seen.

Management of tuberculous pseudoaneurysm includes initiation of anti-tuberculosis drugs and surgical repair. Medical treatment with anti-tuberculosis drugs not only controls the infection, but also reduces the local tissue inflammation of the pseudoaneurysm, thus lowering the risk of operative complications.^7^ Surgical repair of the pseudoaneurysm is imperative due to the high risk of rupture.^7^ The most common surgical approach involves resection of the diseased segment, removal of the surrounding necrotic tissues, and reconstruction of the vessel using a graft.^8^ A recent case report by Gamble described a patient with rapidly expanding tuberculous pseudoaneurysm of the infra-renal aorta after hematogenous spread from intravesical BCG chemotherapy for bladder cancer that was treated successfully with open surgery, local debridement of mycotic pseudoaneurysm, in-situ surgical reconstruction using a custom bovine-wrap interposition graft, and multi-agent anti-tuberculosis chemotherapy.^9^ Although this represents an extremely rare complication from intravesical BCG therapy, this case report aligns with our own experience managing tuberculous pseudoaneurysm in the pursuit of timely open surgical treatment.

Endovascular aneurysm repair (EVAR) is also another treatment option of pseudoaneurysms first introduced in the 1990s, with lower perioperative morbidity and mortality compared to open repair.^10^ However, a prior literature review examining cases of endovascular repaired tuberculous aortic aneurysms found a risk for prosthetic or graft infection.^8^ In our patient, the kidney was non-functional and open surgical resection was necessary due to the enlarging pseudoaneurysm and high risk of hemorrhage and infectious spread, involvement of the IVC, and complex reconstruction.

## 4 Conclusions

This case demonstrates the most contemporary case of tuberculous pseudoaneurysm of the renal artery in the literature in almost 50 years. It is important for clinicians to recognize this rare manifestation of extra-pulmonary tuberculosis and manage patients with expeditious anti-tuberculous therapy with plans for surgical repair as soon as medically feasible.

## Sources of funding

None.

## CRediT authorship contribution statement

**Sikai Song:** Writing – review & editing, Writing – original draft, Data curation. **Martin Hofmann:** Writing – review & editing. **Herbert Ruckle:** Writing – review & editing. **Forrest Jellison:** Writing – review & editing. **Muhannad Alsyouf:** Writing – review & editing, Conceptualization.

## Declaration of competing interest

None.
